# Curcumin chitosan microspheres regulate Th17/Treg balance via IGF2BP1- mediated m6A modification of LRP5 in ulcerative colitis

**DOI:** 10.22038/ijbms.2024.76332.16535

**Published:** 2024

**Authors:** Ling Chen, Yanru Xiang, Shirong Zhong, Yinglin Wu, Jiaqi Liu, Yan Wu, Zhizhi Wang, Guodong Huang

**Affiliations:** 1 Traditional Chinese Medicine Department, Jiangxi Provincial People’s Hospital, The First Affiliated Hospital of Nanchang Medical College, Nanchang, Jiangxi, China; 2 Anorectal Department of Integrated Traditional Chinese and Western Medicine, The First Affiliated Hospital, Jiangxi Medical College, Nanchang University, Nanchang, Jiangxi, China; 3 Department of Rehabilitation Medicine, Jiangxi Provincial People’s Hospital, The First Affiliated Hospital of Nanchang Medical College, Nanchang, Jiangxi, China; 4 Department of Anesthesiology, The First Affiliated Hospital, Jiangxi Medical College, Nanchang University, Nanchang, Jiangxi, China; 5 Department of Ophthalmology, The First Affiliated Hospital, Jiangxi Medical College, Nanchang University, Nanchang, Jiangxi, China; 6 Digestive System Department, The First Affiliated Hospital, Jiangxi Medical College, Nanchang University, Nanchang, Jiangxi, China; # These authors contributed equally to this work

**Keywords:** Inflammatory bowel disease, Medicine, N6-methyladenosine- modification, Th17 cell, Treg cell

## Abstract

**Objective(s):**

Ulcerative colitis (UC) is a commonly recurrent inflammatory bowel disease. T helper 17 (Th17)/regulatory T (Treg) cell balance plays an essential role in UC progression. However, it is unknown whether curcumin chitosan microspheres (CCM) regulate the Th17/Treg cell balance.

**Materials and Methods:**

The UC mouse model was established by administering 3% dextran sodium sulfate and treated with CCM. The influence of CCM on the Th17/Treg balance was detected using flow cytometry. Cell experiments were conducted to investigate the role and mechanism of IGF2BP1 in Th17/Treg balance.

**Results:**

We revealed that CCM demonstrated a significant therapeutic effect on UC. CCM obviously decreased the Th17 cell percentage but boosted the Treg cell percentage in UC mice. CCM remarkably increased the mRNA expression of Foxp3 but suppressed RORγt and interleukin-10 mRNA expression. PCR array of RNA modification-related genes revealed that the m6A binding protein IGF2BP1 was a key molecule in CCM regulation of Th17/Treg balance. IGF2BP1 overexpression dramatically repressed the CCM-induced balance of Th17/Treg cell differentiation. Mechanically, IGF2BP1 targeted LRP5 and regulated LRP5 through m6A modification. Furthermore, the silencing of LRP5 canceled the suppressive effect of IGF2BP1 on Th17/Treg cell percentage.

**Conclusion:**

CCM modulated the Th17/Treg balance through IGF2BP1-mediated m6A modification, thereby alleviating UC, and providing new ideas for the treatment of UC.

## Introduction

Ulcerative colitis (UC) is a gastrointestinal disease with chronic recurrent characteristics. The observed clinical features of patients with UC include spread mucosal inflammation in the distal colon and rectum, accompanied by rectal bleeding, abdominal pain, diarrhea, and weight loss, which is seriously harmful to human health and increases the risk of developing cancer (1). At present, the traditional treatment for patients with UC mainly includes Western drugs, such as aminosalicylic acid, glucocorticoids, and immunosuppressants. However, problems, such as large side effects, many adverse reactions, high recurrence rate after drug withdrawal, and high price, occur (2, 3). Therefore, developing safer and more efficient medicines for UC treatment is a pressing clinical problem.

Curcumin has wide pharmacological impacts, such as anti-inflammatory, anti-oxidant, lipid-lowering, anticoagulant, antitumor, and scavenging of oxygen free radicals, which is a type of polyphenol extracted from Curcuma longa (4). Curcumin mitigated sodium dextran sulfate-induced colitis by regulating the TLRs signaling pathway and M1/M2 macrophage polarization (5). Gong *et al*. revealed that curcumin alleviated sodium dextran sulfate-induced colitis by restraining interleukin (IL)-1β production and NLRP3 inflammatory body activation (6). However, curcumin has poor stability with low water solubility under acidic and neutral conditions, rapid decomposition under alkaline conditions, and short metabolic half-life *in vivo*, which severely limits its use as a pharmaceutical preparation (7). Hatamipour *et al*. revealed that a novel nanomicellar formulation improved the bioavailability and stability of curcuminoids (8). The analysis of its particle properties, solubility, stability, and pharmacokinetics revealed that nano-micelles significantly improved the water solubility, oral bioavailability, and stability of curcuminoids compared with free curcumins. Additionally, enhanced pharmacokinetic parameters were observed in mouse models, indicating that this novel formulation improved curcumin-like absorption and stability. The drug sustained-release system is one of the most popular fields in drug research in recent years. Drug molecules and drug carriers need to be combined in a certain physical or chemical way to slow down drug release to achieve better therapeutic effects in terms of controlled drug release technology. Microspheres are tiny spherical entities with a diameter of 1-1000 µm and are widely used in drug delivery applications (9). Chitosan is a polymer with various biological characteristics, such as harmlessness, biodegradability, and viscosity (10, 11). Chitosan has good biocompatibility and can be used as a sustained-release carrier to improve drug utilization rate. Chitosan microspheres have important applications in drug delivery systems. Researchers prepared curcumin chitosan microspheres (CCM) and revealed their good antibacterial, anti-oxidant, and anti-inflammatory activities (12). However, the role and molecular mechanism of CCM in UC remains unclear.

Immune microenvironment imbalance is a key factor in UC, in which the T helper 17 cells (Th17)/Regulatory T (Treg) cell imbalance exerts a pivotal part (13). Th17 cells are effector T-cell subsets distributed in the mucous membrane, especially the intestinal mucosa. These cells mainly secrete the inflammatory cytokines IL-21, IL-17A, and IL-22 to regulate the immune response. Th17 cells can not only aggravate the intestinal inflammatory response by proinflammatory cytokines but also protect the intestinal mucosa by maintaining the balance of the immune microenvironment (14). The involvement of Th17 cells has been observed in the pathogenesis of rheumatoid arthritis, psoriasis, and inflammatory bowel disease (15). Treg cells are a Th subgroup that induces autoimmune tolerance and are characterized by CD4^+^ CD25^+ ^Foxp3^+^ (16). Treg cells can protect the host from overactive immune responses and tissue damage (17). Research has shown that Th17/Treg balance plays a crucial role in the occurrence and development of colitis. For example, Yu et al. revealed a higher Th17 cell ratio and a lower Treg cell ratio in the peripheral blood of patients with UC than that of the healthy group (18). A study uncovered that the Th17 cell percentage in UC patients was significantly enhanced, while the percentage of Treg cells was reduced relative to healthy controls (19). Tripterygium wilfordii polyglycoside reduced the severity of TNBS-regulated Th17/Treg balance in the intestinal mucosa to induce colitis in rats (20). These studies indicate that the tradeoff between Th17 and Treg is a crucial step in UC progression.

The m6A modification is an RNA epigenetic modification that occurs during transcription (21). It involves three functional proteins: “Writer,” “Erasers,” and “Reader.” Evidence indicates that m6A has different regulators that play a crucial role in the m6A modification of inflammation, various tumors, innate immunity, and immunotherapy (22). A study found that the loss of the m6A recognition protein promoted the development of dextran sodium sulfate (DSS)-induced colitis in mice (23). Furthermore, the m6A modification is relevant to immune infiltration. A lack of METTL14 in T cells promoted spontaneous colitis in mice (24). Tong *et al*. uncovered that severe autoimmune diseases occurred in mice after the specific knockdown of METTL3 in Treg cells (25). Researchers discovered that ALKBH5 reduced m6A modifications of CXCL2 and interferon (IFN)-γ mRNA, and augmented protein expression and transcriptional stability, thereby promoting CD4^+^ T cell response during neuroinflammation (26). However, the moderating role of m6A modification on the Th17/Treg balance in UC has not been reported.

In the present study, we aimed to probe the function and mechanism of CCM in Th17/Treg balance in UC mice. The effect of CCM on UC and Th17/Treg balance was evaluated by animal experiments. PCR array was used to identify the key molecules involved in the Th17/Treg cell balance regulated by CCM. The effect of IGF2BP1 on Th17/Treg cell balance was then investigated. Subsequently, bioinformatics analysis was used to identify the target gene of IGF2BP1. Finally, rescue experiments were conducted. Our study provides novel insights into the mechanism of CCM treating UC.

## Materials and Methods

***CCM preparation***

CCM was prepared using the ion crosslinking method. In detail, chitosan was dissolved in 1% acetic acid to prepare a chitosan mixture with a 10 g/l concentration. Curcumin was then dissolved in diethylene glycol ether to prepare a curcumin mixture with a 10 g/l concentration. The above two solutions were mixed at a ratio of 0.83 in volume and ultrasonically degassed for use. Subsequently, sodium tripolyphosphate was dissolved in distilled water to form a solution with a concentration of 0.15% sodium tripolyphosphate. The same volume of sodium tripolyphosphate was gradually dropped into the mixture of curcumin and chitosan under magnetic force stirring. After stirring the mixtures for 10 min, centrifugation was conducted three times at 6000 rpm for 4 min. Finally, the obtained precipitate, namely CCM, was washed three times in purified water, and the collected precipitate was freeze-dried and stored in a dryer for later use. 

***Experimental animal and treatment***

We obtained BALB/c mice (male, 6-week-old) from SPF Biotechnology Co., Ltd (Beijing, China). The mice then resided in a standard environment (temperature: 24 ^°^C ± 2 ^°^C, humidity: 55%±10%, with a 12 hr light/dark cycle) and received water and food freely. A mouse model of UC was constructed by administering mice 3% DSS for 7 days (27). Mice were randomly assigned to three groups: control, model, and model+CCM groups (n=5 per group). The control group was provided with regular water. The model group received 3% DSS. The CCM group was gavaged with 20 g/l/kg CCM once a day for 10 continuous days after model induction.

***Assessment of the UC***

The body weight, stool characteristics, and bloody stool of mice were monitored daily, and the disease activity (DAI) score was calculated as previously reported (28). The colon, lymph nodes, and spleen were collected, and the severity of colitis was further assessed by morphological changes of organs. The spleen, lymph nodes, and colon were weighed, and the colon length from the cecum to the rectum was measured. The histological injury score was determined using the criteria developed by Dieleman *et al*. (29). Colon tissue samples were collected and hematoxylin and eosin (HE) staining were used to analyze the pathological variations of the colon. Histopathological scores were obtained following the report of Dong *et al*. (27).

***Flow cytometry***

The spleen tissue of mice was removed, and CD4^+^T cells were isolated. Cells were resuspended in RPMI-1640 medium (Sangon, China). To detect Th17 cells, cells were incubated with 1 μl Cell Stimulation cocktail (eBiosciences, USA) at 37 ^°^C for 4 hr. FITC-labeled anti-CD4 and APC-labeled anti-IL-17A antibodies were used to stain cells. FITC-labeled anti-CD4, PE-labeled anti-CD25 and APC-labeled anti-Foxp3 antibodies were used to detect Treg cells. Finally, cells were tested by flow cytometry (BDBioscience).

***Quantitative real-time PCR (qRT*** ***PCR)***

We isolated total RNA from cells using TRIzol reagent (Invitrogen) following the manufacturer’s instructions. The concentration and purity of RNA were observed using a microspectrophotometer (TIANGEN). Reverse transcription was then implemented using the RevertAid First Strand cDNA Synthesis Kit (K1622, Thermo). Afterward, qRT–PCR was conducted using 2×Master Mix (Roche) on an ABI QuantStudio 6 Flex Real-Time PCR system (Applied Biosystems Inc., USA). The 2^−^^ΔΔCT^ method was applied to detect RORγt, Foxp3, IL10, IGF2BP1, CPSF3, YTHDC1, YTHDF1, HNRNPC, RPTOR, LRP5, PIK3CD, MAP2K2, and WDR24 mRNA expression. Table S1 lists the primer sequences. Supplementary Information File 1 shows the confirmation of the primer blast from NCBI.

***Isolation and activation of CD4*** ^+^ ***T cells and CCM treatment***

The mouse spleen was collected to acquire single cell suspensions, and CD4^+ ^T cells were separated using EasySep™ Mouse CD4^+ ^T-Cell Isolation Kit (Stemcell Technologies) following the manufacturer’s manual. Subsequently, 10 µg/ml of anti-CD3 and 5 µg/ml of anti-CD28 were appended to activate CD4^+ ^T cells. CD4^+^ T cells were appended with IL-2 (2 ng/ml) and TGF-β1 (5 ng/ml) to trigger Treg cell differentiation, and treated with TGF-β1 (5 ng/ml), IL-2 (5 ng/ml), IL-6 (50 ng/ml), anti-IL-IFN-γ antibody (5 µg/ml), and anti-IL-4 antibody (5 µg/ml) to trigger Th17 cell differentiation. Subsequently, CD4^+^ T cells were dealt with 25 μM of CCM for 24 hr according to the previous report (30).

***PCR array of RNA epigenetic modification-related genes***

RNA extraction was conducted using the RNeasy Min kit (Qiagen, Germany). NanoDrop®ND-1000 and agarose gels were used to determine concentration and purity. RNA was reverse transcribed into cDNA using the RT^2^ First Strand Kit (Qiagen, Germany). The qRT-PCR was conducted with the RT2 SYBR Green Mastermix (Qiagen, Germany) and PCR Array. The relative expression of RNA epigenetic modification-related genes was calculated by the 2^−ΔΔCt ^approach.

***Immunohistochemistry***

The colon tissue samples were fixed in 4% formaldehyde, paraffin-embedded, and subsequently sliced into 4 μm sections. The sections were subjected to dewaxing, rehydration, and antigen retrieval. Subsequently, the sections were incubated with an anti-IGF2BP1 antibody (1/4000, ab184305, abcam) and an HRP-conjugated secondary antibody anti-IgG (CLOUD-CLONE CORP., Wuhan, China). Images were observed under a microscope (OLYMPUS CK31).

***Gene set enrichment analysis (GSEA)***

GSE161087 was utilized to perform GSEA. We set the threshold value as *P*-value of <0.05 and false discovery rate of <0.01.

***Cell transfection***

The mRNA of IGF2BP1 was cloned into pcDNA3.1 (+) plasmid to construct the overexpressed IGF2BP1 vectors. IGF2BP1 overexpressed vector plasmid and negative control vector plasmid were transfected into CD4^+ ^T cells using Lipofectamine™ 3000 (Invitrogen, California, USA) for 24 hr following the manufacturer’s instructions. Small interfering RNAs (siRNAs) targeting LRP5 (si-LRP5) were transfected into CD4^+^ T cells to knock down LRP5.

***Prediction of the interaction between IGF2BP1 and LRP5 and m6A modification***

The eCLIP-seq database was used to predict the association between IGF2BP1 and LRP5. The Rmbase and SRAMP databases were used to unmask the m6A modification of LRP5.

***RNA immunoprecipitation (RIP) assay***

To ascertain the relation between IGF2BP1 and LRP5, the Magna RIP RNA protein immunoprecipitation kit (Millipore) was applied for the RIP assay. CD4^+ ^T cells were lysed using RNA lysis buffer and then cultivated with the RIP Wash buffer comprising magnetic beads coated with anti-IGF2BP1 or IgG antibodies. After the magnetic beads were rinsed with RIP wash buffer, the proteins were digested using proteinase K. The precipitated RNAs were isolated and purified for qRT–PCR analysis.

***m*** ^6^ ***A methylated RNA immunoprecipitation-PCR (MeRIP-PCR)***

MeRIP-PCR analysis was performed to evaluate the m6A level of LRP5. Briefly, the cells were collected and total RNA was extracted. RNA fragments were incubated with A/G magnetic beads containing anti-m6A or IgG antibodies after sonication. After MeRIP, LRP5 mRNA was detected by qRT–PCR.

***Western blot***

Proteins from CD4^+ ^T cell samples were extracted with RIPA buffer (Thermo). Proteins were segregated by SDS-PAGE and then transferred to polyvinylidene fluoride membranes. The membranes were incubated with anti-IGF2BP1 (1/1000, 22803-1-AP, Proteintech), anti-LRP5 (1/1000, 24899-1-AP, Proteintech), anti-RORγt (1/1000, Abcam), anti-Foxp3 (1/1000, 22228-1-AP, Proteintech), anti-IL-10 (1/1000, Bs-0698R, Bioss), and anti-GAPDH (1/2000, 60004-1-Lg, Proteintech) primary antibodies after blocking with 5% skim milk. Finally, a secondary antibody, Goat Anti-Mouse IgG H&L (HRP) (1/1000, ab205719, Abcam), was used to incubate the membranes, and ECL reagents (Thermo) were used for color development.

***Statistical analysis***

GraphPad Prism 9 software was used for statistical analysis. Data are presented as mean±standard deviation. Student’s *t*-test was adopted to assess differences between the two groups, and one-way analysis of variance was applied to evaluate differences between more than two groups. A *P*-value of <0.05 indicated statistical significance.

## Results

***Curcumin chitosan microspheres alleviate UC in mice***

**Figure 1:**
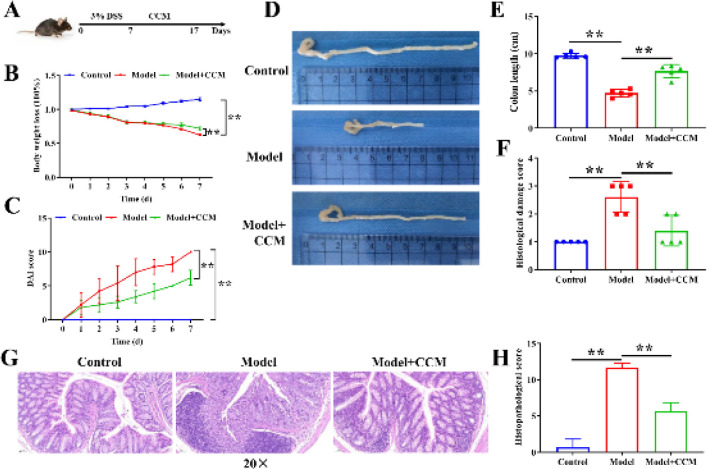
Curcumin chitosan microspheres (CCM) mitigate ulcerative colitis (UC) in mice

In order to investigate the effects of CCM on UC, we first prepared CCM. CCM were spherical, had a smooth surface with a diameter of approximately 2 µm, and had good dispersion. The polymer dispersity index of CCM was 0.28, and the encapsulation rate was 75.11%. Subsequently, the UC mouse model was constructed by DSS and treated with CCM (Figure 1A). We found that DSS treatment resulted in a remarkable weight loss, a significant increase in DAI score, colon shortening, and a significant increase in histological damage score, whereas CCM reversed these changes (Figure 1B-1F). HE staining disclosed that the colon tissue structure was regular and glands were neatly arranged in the control mice. The colon mucosa of UC mice was severely damaged the mucosal glands were disordered and deformed inflammatory cell infiltration increased. As expected, the mucosa was relatively intact, and inflammatory cell infiltration was lessened after CCM administration (Figure 1G). Meanwhile, CCM treatment reduced the increased histopathological score in UC mice (Figure 1H). Altogether, CCM ameliorate colon injury in UC mice.

***Curcumin chitosan microspheres promote Th17/Treg cell balance***

**Figure 2:**
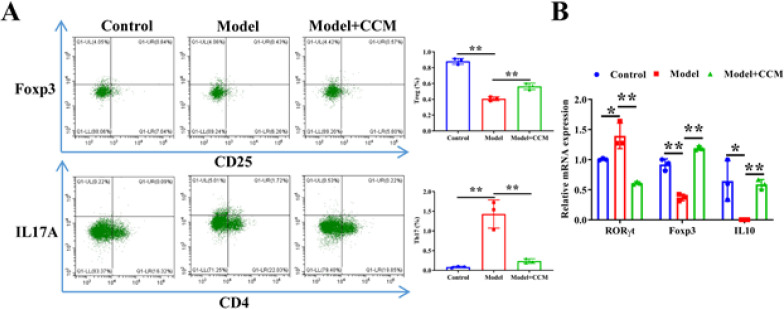
Curcumin chitosan microspheres (CCM) promote Treg cell differentiation in UC mice

We assessed the proportion of Th17 and Treg cells to determine whether CCM regulate the Th17/Treg cell balance. Flow cytometry analysis showed that DSS treatment prominently increased the Th17 cell ratio and reduced the Treg cell ratio in the spleen tissues of mice, whereas these trends were reversed after CCM intervention (Figure 2A). qRT-PCR assay uncovered that DSS treatment remarkably increased RORγt mRNA expression in the spleen tissue of mice, whereas it lessened mRNA expression of IL-10 and Foxp3. Inversely, their trends were reversed after CCM treatment (Figure 2B). These results suggest that CCM regulate the Th17/Treg cell balance.

***Curcumin chitosan microspheres modulate Th17/Treg cell balance through IGF2BP1***

**Figure 3:**
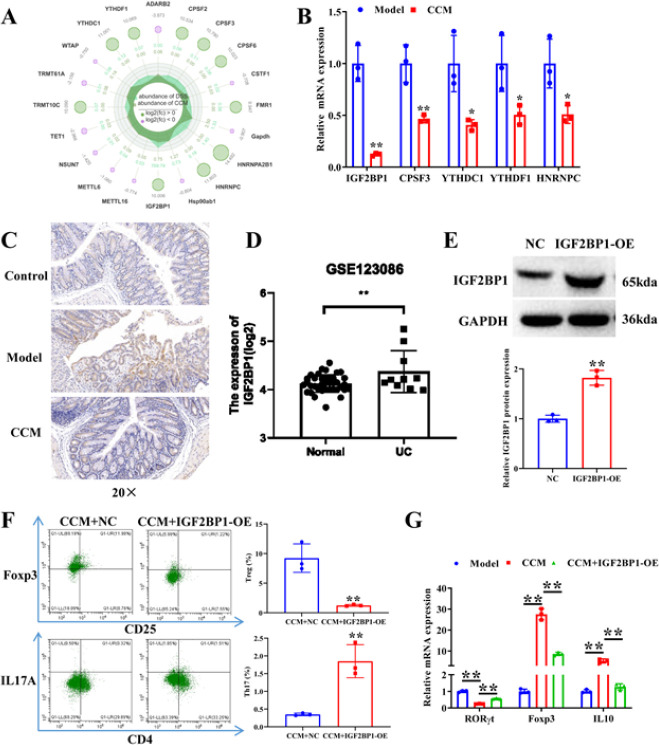
Curcumin chitosan microspheres (CCM) adjust Th17/Treg balance through IGF2BP1 in UC mice

To identify the key molecules that participate in the mediation of Th17/Treg cell balance by CCM, CD4^+^ T cells were separated from the spleen tissues of mice in the model and CCM groups for PCR array detection. A total of 84 genes that correlated to RNA modification were screened (Figure 3A, Table S2). We selected five genes with high expression as candidate molecules. qRT-PCR verification showed that IGF2BP1 significantly declined in the CCM group relative to the model group with the largest fold change (Figure 3B). Therefore, IGF2BP1 was selected for subsequent research. Immunohistochemistry analysis revealed that the expression of IGF2BP1 was raised in UC mice, whereas CCM lessened IGF2BP1 expression in UC mice (Figure 3C). Database GSE123086 analysis also showed that IGF2BP1 was remarkably up-regulated in UC (Figure 3D). We then investigated the effect of IGF2BP1 on Th17/Treg cell balance. Western blot verified the overexpression efficiency of IGF2BP1 (Figure 3E). Overexpression of IGF2BP1 remarkably increased the ratio of Th17 cells, while reducing the Treg cells differentiated from CD4^+ ^T cells treated with CCM, as demonstrated by flow cytometry (Figure 3F). IGF2BP1 overexpression recovered the inhibitory effect of CCM on RORγt and inhibited Foxp3 and IL-10 up-regulation induced by CCM, as confirmed by qRT–PCR (Figure 3G). All the results above suggest that CCM regulate Th17/Treg cell balance through IGF2BP1.

***IGF2BP1 adjusts Th17/Treg cell balance through the LRP5-mediated mTOR signaling pathway***

**Figure 4:**
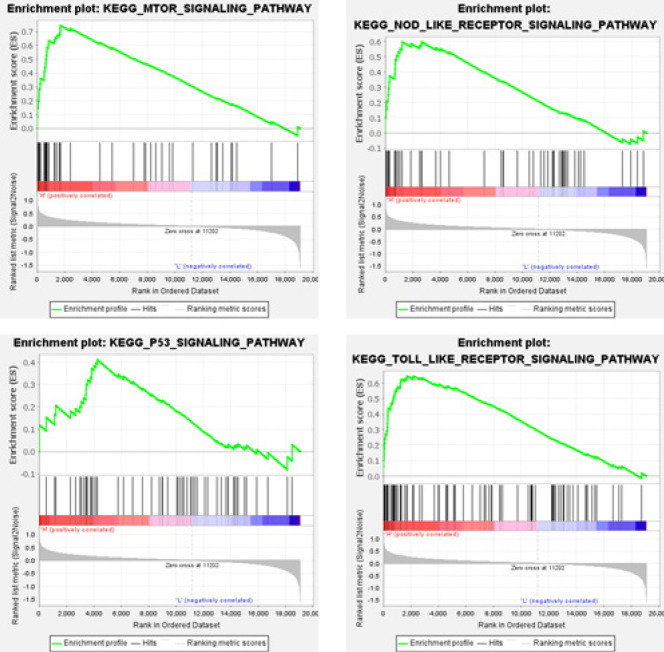
Gene set enrichment analysis (GSEA) enrichment analysis of the GSE123086 dataset

**Figure 5:**
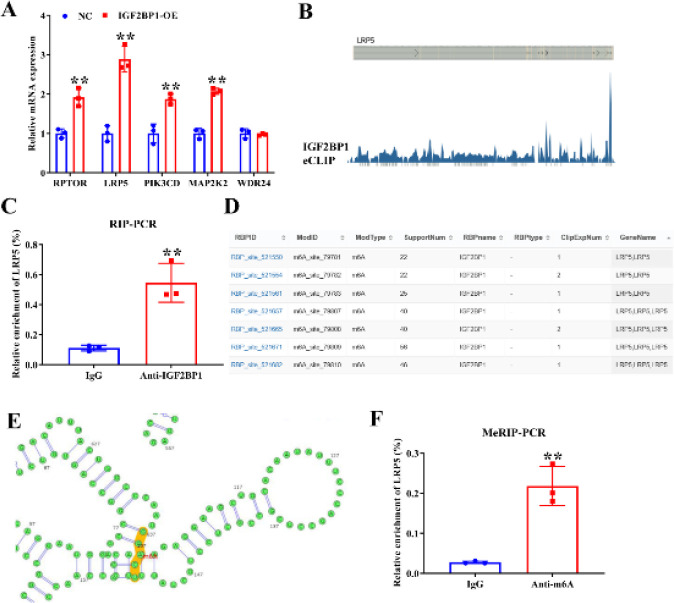
IGF2BP1 regulates LRP5 expression

To investigate the downstream pathways regulated by IGF2BP1, we divided the UC samples from the GSE123086 database into two groups based on high and low expression levels of IGF2BP1. Subsequently, we performed GSEA to analyze the enrichment of pathways. The results revealed that IGF2BP1 was involved in the mTOR, NOD_LIKE_RECEPTOR, P53, and TOLL_LIKE_RECEPTOR signaling pathways (Figure 4). A previous study revealed that the mTOR signaling pathway is related to Th17/Treg cell balance (31). Therefore, we next focused on the mTOR signaling pathway. We screened five molecules enriched in the mTOR signaling pathway with high expression. qRT–PCR verification revealed that LRP5 was dramatically up-regulated after IGF2BP1 overexpression, with the largest fold change (Figure 5A). Therefore, LRP5 was selected as the target gene. The eCLIP-seq database analysis revealed that IGF2BP1 is bound to LRP5 mRNA (Figure 5B). RIP-PCR results further confirmed that IGF2BP1 interacted with LRP5 (Figure 5C). The Rmbase database demonstrated that m6A modification occurred at IGF2BP1 binding sites on LRP5 mRNA (Figure 5D). The m6A modification of LRP5 mRNA was further analyzed using the SRAMP database (Figure 5E). MeRIP-PCR verified the m6A modification of LRP5 mRNA (Figure 5F). These results suggest that IGF2BP1 may regulate LRP5 through m6A modification.

***IGF2BP1 regulates Th17/Treg cell balance by mediating LRP5 expression***

**Figure 6:**
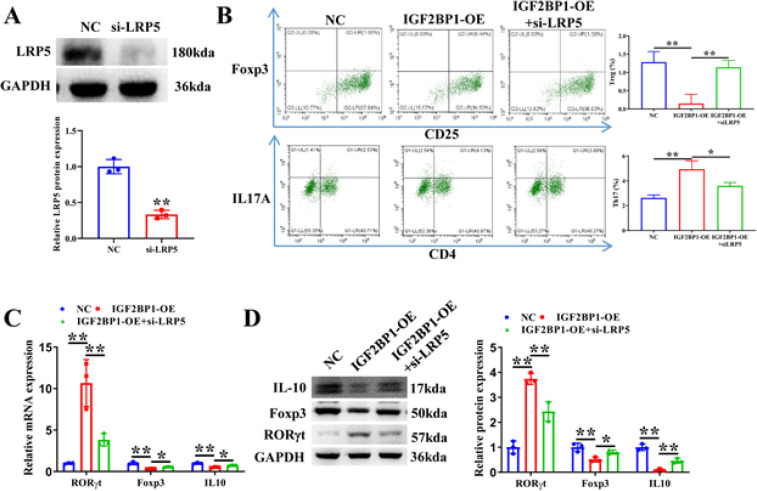
LRP5 is implicated in the modulation of Th17/Treg cell balance by IGF2BP1

To investigate whether IGF2BP1 regulates Th17/Treg cell balance by LRP5, IGF2BP1 overexpression vector and si-LRP5 were transfected in CD4^+^ T cells. Western blot verified the knockdown efficiency of LRP5 (Figure 6A). Flow cytometry analysis revealed that IGF2BP1 overexpression notably boosted Th17 cell percentage and reduced Treg cell percentage, whereas LRP5 knockdown reversed this trend (Figure 6B). qRT-PCR and western blot assays revealed that LRP5 depletion canceled the promoting influence of IGF2BP1 overexpression on RORγt expression and the down-regulation of Foxp3 and IL-10 expression by IGF2BP1 overexpression (Figure 6C, 6D). Therefore, IGF2BP1 regulates Th17/Treg cell balance by up-regulating LRP5 expression.

(A) Schematic diagram of animal experimental design. The mice were allocated to three groups (control, model, and model+CCM) with 5 mice in each group. (B) Body weight changes of each group. (C) Impact of CCM on DAI score. (D) Typical images of colon length. (E) Analysis of the length of colons of each group. (F) Histological damage scores of colon tissues. (G) Hematoxylin and eosin (HE) staining of colon tissue sections of CCM-treated UC mice. (H) Histopathological scores of colon tissues. Data are exhibited as mean±standard deviation (SD). **P<0.01

(A) Flow cytometry analysis of Treg and Th17 cells in the spleen tissues of UC mice treated with CCM. (B) Expression of Th17 and Treg cell markers in spleen tissues was assayed by qRT–PCR. Data are exhibited as mean±SD. *P<0.05, **P<0.01.

(A) Key molecules involved in the regulation of Th17/Treg balance were screened by PCR array of CD4^+^ T cells derived from the spleen tissue of the model and CCM groups. (B) Expression of candidate m6A modification-related genes was assayed by qRT-PCR. (C) Expression of IGF2BP1 was ascertained by immunohistochemistry. (D) The expression of IGF2BP1 in patients with UC was determined by the GEO database GSE123086. (E) Western blot analysis of the overexpression efficiency of IGF2BP1. (F) Flow-cytometry analysis of Treg and Th17 cell ratios upon IGF2BP1 overexpression. (G) Expression of Th17 and Treg cell markers after CCM treatment and IGF2BP1 overexpression was examined by qRT-PCR. Data are exhibited as mean±SD. * *P<*0.05, ** *P<*0.01

NC: normal control; IGF2BP1-OE: IGF2BP1 overexpression

The ulcerative colitis (UC) samples were divided into two groups on the basic of high and low expression levels of IGF2BP1, followed by GSEA analysis

(A) Expression of candidate target genes of IGF2BP1 was tested using qRT-PCR. (B) eCLIP-Seq database analysis revealed that IGF2BP1 is bound with LRP5 mRNA. (C) Binding of IGF2BP1 and LRP5 was detected by RIP-PCR. (D) Rmbase database showed that m6A modification occurred at IGF2BP1 binding site on LRP5 mRNA. (E) m6A modification of LRP5 mRNA was analyzed by SRAMP database. The figure showed the secondary structure of the site with the highest score. (F) m6A modification of LRP5 mRNA was verified by MeRIP-PCR. Data are exhibited as mean±SD. *P<0.05, **P<0.01

(A) Flow cytometry analysis of the proportion of Treg and Th17 cells upon IGF2BP1 overexpression and LRP5 knockdown. (B) Knockdown efficiency of LRP5 was confirmed by western blot. (C) qRT–PCR and (D) western blot analysis of the expression of Th17 and Treg cell markers in IGF2BP1 overexpressed CD4^+^ T cells transfected with LRP5 siRNA

**Figure 7:**
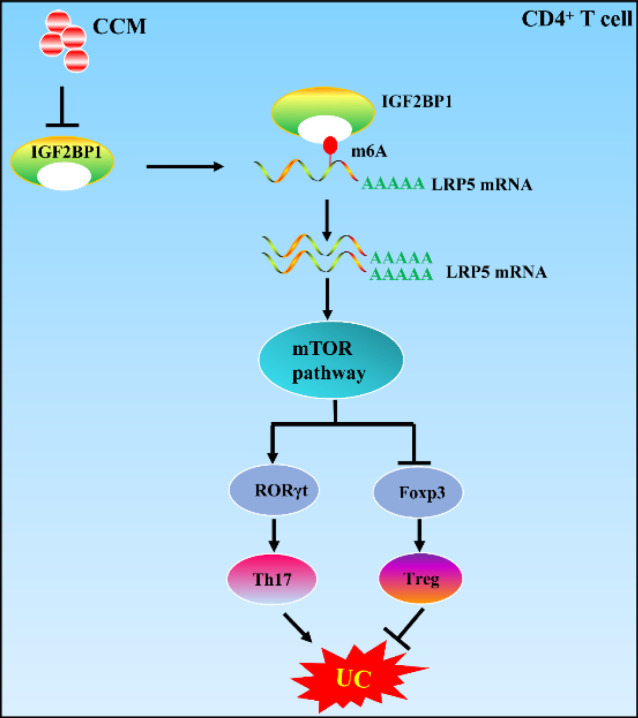
Schematic representation of the role and mechanism of curcumin chitosan microspheres (CCM) in ulcerative colitis (UC)

## Discussion

UC is a chronic inflammatory disease, and its incidence is increasing worldwide. Therefore, it is important to understand the pathogenesis of UC and find optimal treatment for UC. In this study, we discovered that CCM had a significant therapeutic effect on UC and restored the Treg/Th17 balance. PCR array of RNA modification suggested that IGF2BP1 was implicated in the CCM regulation of the Th17/Treg balance. IGF2BP1 overexpression inhibited CCM-induced Treg cell differentiation. Mechanically, IGF2BP1 targeted LRP5 and modulated LRP5 via m6A modification (Figure 7).

Th17 and Treg cells are two forms of CD4^+^ T cell subsets and are crucial in maintaining the immune balance. RORγt is a specific transcription factor that promotes Th17 cell differentiation. It induces Th17 cells to secrete IL-17 and other proinflammatory factors, thereby contributing to intestinal inflammation (32). Foxp3 is a characteristic nuclear transcription factor of Treg cells, which regulates Treg cell differentiation and leads to the production of IL-10 and other anti-inflammatory factors (33). They maintain dynamic balance and jointly maintain immune homeostasis. Th17/Treg imbalance is implicated in various inflammatory diseases such as multiple sclerosis, rheumatoid arthritis, and inflammatory bowel disease (34-36). Th17/Treg imbalance is also associated with colitis. Astragaloside IV ameliorated UC by regulating Th17/Treg cell homeostasis (37). Total flavonoids of *Sophora flavescens* and kurarinone alleviated UC via modulating the Th17/Treg balance (38). In agreement with these findings, we discovered that CCM prominently lessened the ratio of Th17 cells and enhanced the ratio of Treg cells in UC mice. CCM also notably up-regulated the Foxp3 and IL-10 mRNA expression, whereas it down-regulated RORγt mRNA expression in UC mice. These results reveal that CCM treatment improves Treg/Th17 balance.

Recently, m6A regulators have been found to be related with immune cell infiltration. METTL3-mediated m6A methylation facilitates dendritic cell function in T-cell activation (39). M6A modification regulates the naive T cell differentiation by targeting SOCS mRNA, thereby maintaining the homeostasis of the immune system (40). Overexpression of m6A writer WTAP contributes to the Treg cell differentiation and the augments of Treg cell-mediated repression to naïve T cells (41). ALKBH5 modulated CD4^+^ T cells to respond and enhance autoimmunity (26). In the present study, IGF2BP1 facilitated Treg cell differentiation and restrained Th17 cell differentiation, indicating that IGF2BP1 modulated the Th17/Treg balance in UC.

The mTOR signaling pathway plays a crucial role in Th17/Treg balance. mTOR inhibition in CD4^+^ T cells promotes Treg differentiation and is conducive to renal protection (31). mTOR suppression alleviates DSS-induced colitis by balancing the TH1/TH17/Treg profile (42). Repression of the mTOR signaling pathway restores Th17/Treg balance in asthmatic mice (43). In the present study, we discovered that IGF2BP1 targeted LRP5 which was involved in the mTOR signaling pathway, and IGF2BP1 regulated LRP5 through m6A modification. Therefore, IGF2BP1 participates in the mTOR signaling pathway by regulating LRP5 expression through m6A modification and then regulates Th17/Treg balance.

## Conclusions

In conclusion, CCM regulate Th17/Treg balance through IGF2BP1-mediated m6A modification of LRP5 in UC. The present research provides novel insight into the mechanism of CCM in UC treatment.

## Data Availability

The datasets used and/or analyzed during the current study are available from the corresponding author upon reasonable request.

## Supplementary Table

**Table S1:** Primer sequences

Name	Sequences (5' to 3')
m-Actin-F	CCACCATGTACCCAGGCATT
m-Actin-R	AGGGTGTAAAACGCAGCTCA
mmu- RORγt-F	CGCGGAGCAGACACACTTA
mmu- RORγt-R	CCCTGGACCTCTGTTTTGGC
mmu-Foxp3-F	ACCATTGGTTTACTCGCATGT
mmu-Foxp3-R	TCCACTCGCACAAAGCACTT
mmu-IL10-F	AGCCTTATCGGAAATGATCCAG
mmu-IL10-R	GGCCTTGTAGACACCTTGGT
mmu-IGF2BP1-F	CGCTATCATTGGCAAGGAGG
mmu-IGF2BP1-R	GTGTCCTTTGCCTCCTTGTG
mmu-CPSF3-F	CAGCAACATATCGGCAGATGA
mmu-CPSF3-R	CTTCACACCAGCGATCTCTATC
mmu-YTHDC1-F	GTCCACATTGCCTGTAAATGAGA
mmu-YTHDC1-R	GGAAGCACCCAGTGTATAGGA
mmu-YTHDF1-F	CCCCAGAGAACAAAAGGACA
mmu-YTHDF1-R	TCGCTGAGGGAGTAAGGAAA
mmu-HNRNPC-F	CGAAGAGTGGACAAAGGGGA
mmu-HNRNPC-R	CTGGGGATGAGAAGGACAAGT
mmu-RPTOR-F	CAGCCCATTCTTTGCCGAG
mmu-RPTOR-R	CGTCCCAGCAAGTCCAATG
mmu-LRP5-F	GAACATTGTCATCTCGGGCC
mmu-LRP5-R	GTCCTGCCAGAAGAGAACCT
mmu-PIK3CD-F	GGCCAACCTCATGCTATTCG
mmu-PIK3CD-R	TTCAGCAGCTCTCCCTTCTC
mmu-MAP2K2-F	ACTACTCTGTGCAGTCGGAC
mmu-MAP2K2-R	CTGTCCATCCCATGACCACT
mmu-WDR24-F	TCTGTCAGCACCTTCTCTGG
mmu-WDR24-R	CAGAAGACAGGCCCATTGTG

**Table S2:** PCR array results

Symbol	AVG ΔC_t_ (Ct(GOI) - Ave Ct (HKG))		2^-ΔC_t_		Fold Change	T-TEST
	CCM	Control	CCM	Control	CCM/Control	p value
ADAR	8.70	4.63	2.4E-03	4.0E-02	0.06	N/A
ADARB1	14.01	10.04	6.1E-05	9.5E-04	0.06	N/A
ADARB2	4.02	7.89	6.2E-02	4.2E-03	14.65	N/A
ALKBH1	14.01	4.94	6.1E-05	3.3E-02	0.00	N/A
ALKBH3	7.34	6.84	6.2E-03	8.7E-03	0.71	N/A
ALKBH5	8.92	4.43	2.1E-03	4.7E-02	0.04	N/A
ALYREF	9.96	7.02	1.0E-03	7.7E-03	0.13	N/A
CFI	6.87	6.78	8.6E-03	9.1E-03	0.94	N/A
CLP1	5.20	4.72	2.7E-02	3.8E-02	0.72	N/A
CMTR1	10.51	5.74	6.8E-04	1.9E-02	0.04	N/A
CPSF1	12.67	5.92	1.5E-04	1.7E-02	0.01	N/A
CPSF2	14.01	3.48	6.1E-05	9.0E-02	0.00	N/A
CPSF3	14.01	3.22	6.1E-05	1.1E-01	0.00	N/A
CPSF4	14.01	14.27	6.1E-05	5.1E-05	1.20	N/A
CPSF6	14.01	3.99	6.1E-05	6.3E-02	0.00	N/A
CPSF7	4.97	4.38	3.2E-02	4.8E-02	0.67	N/A
CSTF1	5.18	5.89	2.8E-02	1.7E-02	1.63	N/A
CSTF2	11.64	5.55	3.1E-04	2.1E-02	0.01	N/A
CSTF3	14.01	4.95	6.1E-05	3.2E-02	0.00	N/A
DNMT1	14.01	4.55	6.1E-05	4.3E-02	0.00	N/A
DNMT2	14.01	7.99	6.1E-05	3.9E-03	0.02	N/A
DNMT3A	10.15	5.48	8.8E-04	2.2E-02	0.04	N/A
DNMT3B	12.94	7.67	1.3E-04	4.9E-03	0.03	N/A
eIF3	4.98	1.25	3.2E-02	4.2E-01	0.08	N/A
FBL	4.46	1.95	4.5E-02	2.6E-01	0.18	N/A
FIP1L1	14.01	5.03	6.1E-05	3.1E-02	0.00	N/A
FMR1	14.01	4.06	6.1E-05	6.0E-02	0.00	N/A
FTO	4.62	4.14	4.1E-02	5.7E-02	0.72	N/A
HNRNPA2B1	14.01	-0.48	6.1E-05	1.4E+00	0.00	N/A
HNRNPC	14.01	2.41	6.1E-05	1.9E-01	0.00	N/A
HuR	9.74	4.11	1.2E-03	5.8E-02	0.02	N/A
IGF2BP1	0.42	-9.59	7.5E-01	7.7E+02	0.00	N/A
IGF2BP2	14.01	8.40	6.1E-05	3.0E-03	0.02	N/A
IGF2BP3	12.20	10.10	2.1E-04	9.1E-04	0.23	N/A
LRPPRC	9.44	4.38	1.4E-03	4.8E-02	0.03	N/A
METTL1	14.01	7.29	6.1E-05	6.4E-03	0.01	N/A
METTL14	12.59	6.27	1.6E-04	1.3E-02	0.01	N/A
METTL16	4.22	4.99	5.4E-02	3.1E-02	1.71	N/A
METTL2	8.96	6.13	2.0E-03	1.4E-02	0.14	N/A
METTL3	7.40	3.90	5.9E-03	6.7E-02	0.09	N/A
METTL6	3.54	4.60	8.6E-02	4.1E-02	2.08	N/A
METTL8	10.27	5.40	8.1E-04	2.4E-02	0.03	N/A
NAT10	11.90	5.57	2.6E-04	2.1E-02	0.01	N/A
NML	10.44	5.66	7.2E-04	2.0E-02	0.04	N/A
NOP2	10.58	4.81	6.5E-04	3.6E-02	0.02	N/A
NSUN2	3.79	4.47	7.2E-02	4.5E-02	1.60	N/A
NSUN3	12.52	5.09	1.7E-04	2.9E-02	0.01	N/A
NSUN4	4.70	4.25	3.8E-02	5.3E-02	0.73	N/A
NSUN5	10.45	5.61	7.1E-04	2.1E-02	0.03	N/A
NSUN6	13.21	6.77	1.1E-04	9.2E-03	0.01	N/A
NSUN7	5.59	7.01	2.1E-02	7.8E-03	2.68	N/A
NUDT21	4.67	3.21	3.9E-02	1.1E-01	0.36	N/A
PABPN1	10.61	2.51	6.4E-04	1.8E-01	0.00	N/A
PCF11	12.83	3.57	1.4E-04	8.4E-02	0.00	N/A
PUS1	6.69	5.12	9.7E-03	2.9E-02	0.34	N/A
PUS10	8.54	4.69	2.7E-03	3.9E-02	0.07	N/A
PUS3	14.01	5.00	6.1E-05	3.1E-02	0.00	N/A
PUS7	10.35	6.21	7.6E-04	1.3E-02	0.06	N/A
RBBP6	5.39	3.23	2.4E-02	1.1E-01	0.22	N/A
RBM15	5.44	6.00	2.3E-02	1.6E-02	1.48	N/A
RBM15B	14.01	14.27	6.1E-05	5.1E-05	1.20	N/A
RNMT	5.50	4.88	2.2E-02	3.4E-02	0.65	N/A
SRSF2	2.64	2.12	1.6E-01	2.3E-01	0.70	N/A
TET1	4.96	7.92	3.2E-02	4.1E-03	7.82	N/A
TET2	9.45	5.23	1.4E-03	2.7E-02	0.05	N/A
TET3	11.81	6.27	2.8E-04	1.3E-02	0.02	N/A
THUMPD1	13.51	4.98	8.6E-05	3.2E-02	0.00	N/A
TRDMT1	14.01	7.64	6.1E-05	5.0E-03	0.01	N/A
TRMT10C	14.01	3.92	6.1E-05	6.6E-02	0.00	N/A
TRMT6	14.01	4.99	6.1E-05	3.1E-02	0.00	N/A
TRMT61A	3.97	6.13	6.4E-02	1.4E-02	4.46	N/A
TRUB1	12.36	7.21	1.9E-04	6.8E-03	0.03	N/A
TRUB2	9.96	6.63	1.0E-03	1.0E-02	0.10	N/A
VIRMA	8.13	4.49	3.6E-03	4.5E-02	0.08	N/A
WDR33	4.59	3.40	4.2E-02	9.5E-02	0.44	N/A
WDR4	12.54	5.97	1.7E-04	1.6E-02	0.01	N/A
WTAP	2.81	3.56	1.4E-01	8.5E-02	1.68	N/A
YBX1	1.71	0.58	3.1E-01	6.7E-01	0.46	N/A
YTHDC1	14.01	3.01	6.1E-05	1.2E-01	0.00	N/A
YTHDC2	14.01	6.30	6.1E-05	1.3E-02	0.00	N/A
YTHDF1	14.01	3.94	6.1E-05	6.5E-02	0.00	N/A
YTHDF2	14.01	4.22	6.1E-05	5.4E-02	0.00	N/A
YTHDF3	9.54	3.27	1.3E-03	1.0E-01	0.01	N/A
ZC3H13	14.01	4.12	6.1E-05	5.7E-02	0.00	N/A
Actb	-0.13	-0.20	1.1E+00	1.2E+00	0.95	N/A
B2m	-2.38	-2.86	5.2E+00	7.2E+00	0.72	N/A
Gapdh	-1.32	-0.41	2.5E+00	1.3E+00	1.88	N/A
Gusb	4.17	3.01	5.6E-02	1.2E-01	0.45	N/A
Hsp90ab1	-0.34	0.46	1.3E+00	7.3E-01	1.75	N/A
